# Impact of district mental health care plans on symptom severity and functioning of patients with priority mental health conditions: the Programme for Improving Mental Health Care (PRIME) cohort protocol

**DOI:** 10.1186/s12888-018-1642-x

**Published:** 2018-03-06

**Authors:** Emily C. Baron, Sujit D. Rathod, Charlotte Hanlon, Martin Prince, Abebaw Fedaku, Fred Kigozi, Mark Jordans, Nagendra P. Luitel, Girmay Medhin, Vaibhav Murhar, Juliet Nakku, Vikram Patel, Inge Petersen, One Selohilwe, Rahul Shidhaye, Joshua Ssebunnya, Mark Tomlinson, Crick Lund, Mary De Silva

**Affiliations:** 1 Alan J Flisher Centre for Public Mental Health, Department of Psychiatry and Mental Health, University of Cape Town, 46 Sawkins Road 7700 Rondebosch, Cape Town, South Africa; 2 Department of Population Health, London School of Hygiene and Tropical Medicine, London, UK; 3 College of Health Sciences, School of Medicine, Department of Psychiatry, Addis Ababa University, Addis Ababa, Ethiopia; 4 Centre for Global Mental Health, Health Services and Population Research Department, King’s College London, London, UK; 5 Centre for Innovative Drug Development and Therapeutic Trials for Africa, College of Health Sciences, Addis Ababa University, Addis Ababa, Ethiopia; 6 Global Health and Infection Department, Brighton and Sussex Medical School, University of Sussex, Brighton, UK; 7 Butabika National Referral and Teaching Mental Hospital, Makerere University, Kampala, Uganda; 8 Research and Development Department, HealthNet TPO, Amsterdam, the Netherlands; 9 Center for Global Mental Health, Institute of Psychiatry, Psychology and Neuroscience, King’s College London, London, UK; 10 Research Department, Transcultural Psychosocial Organization (TPO) Nepal, Baluwatar, Kathmandu, Nepal; 11 Aklilu Lemma Institute of Pathobiology, Addis Ababa University, Addis Ababa, Ethiopia; 12 Sangath, Goa, India; 13 Harvard Medical School, Boston, USA; 14 Public Health Foundation of India, New Delhi, India; 15 Centre for Rural Health, School of Nursing and Public Health and School of Applied Human Sciences, University of KwaZulu-Natal, KwaZulu-Natal, South Africa; 16 Centre for Chronic Conditions and Injuries, Public Health Foundation of India, New Delhi, India; 17 CAPHRI School for Public Health and Primary Care, Maastricht University, Maastricht, the Netherlands; 18 Alan J Flisher Centre for Public Mental Health, Department of Psychology, Stellenbosch University, Stellenbosch, South Africa; 19 Department of Population Health, Wellcome Trust, London, UK

**Keywords:** Cohort; depression, Alcohol dependence, Psychosis, Epilepsy, Low-income populations, Primary healthcare

## Abstract

**Background:**

The Programme for Improving Mental Health Care (PRIME) sought to implement mental health care plans (MHCP) for four priority mental disorders (depression, alcohol use disorder, psychosis and epilepsy) into routine primary care in five low- and middle-income country districts. The impact of the MHCPs on disability was evaluated through establishment of priority disorder treatment cohorts. This paper describes the methodology of these PRIME cohorts.

**Methods:**

One cohort for each disorder was recruited across some or all five districts: Sodo (Ethiopia), Sehore (India), Chitwan (Nepal), Dr. Kenneth Kaunda (South Africa) and Kamuli (Uganda), comprising 17 treatment cohorts in total (*N* = 2182). Participants were adults residing in the districts who were eligible to receive mental health treatment according to primary health care staff, trained by PRIME facilitators as per the district MHCP. Patients who screened positive for depression or AUD and who were not given a diagnosis by their clinicians (*N* = 709) were also recruited into comparison cohorts in Ethiopia, India, Nepal and South Africa. Caregivers of patients with epilepsy or psychosis were also recruited (*N* = 953), together with or on behalf of the person with a mental disorder, depending on the district. The target sample size was 200 (depression and AUD), or 150 (psychosis and epilepsy) patients initiating treatment in each recruiting district. Data collection activities were conducted by PRIME research teams. Participants completed follow-up assessments after 3 months (AUD and depression) or 6 months (psychosis and epilepsy), and after 12 months. Primary outcomes were impaired functioning, using the 12-item World Health Organization Disability Assessment Schedule 2.0 (WHODAS), and symptom severity, assessed using the Patient Health Questionnaire (depression), the Alcohol Use Disorder Identification Test (AUD), and number of seizures (epilepsy).

**Discussion:**

Cohort recruitment was a function of the clinical detection rate by primary health care staff, and did not meet all planned targets. The cross-country methodology reflected the pragmatic nature of the PRIME cohorts: while the heterogeneity in methods of recruitment was a consequence of differences in health systems and MHCPs, the use of the WHODAS as primary outcome measure will allow for comparison of functioning recovery across sites and disorders.

## Background

A recent estimation of the global burden of disease indicated that mental, neurological and substance use (MNS) disorders are among the world’s leading causes of disability, accounting for 11.7% of the disability-adjusted life years (DALYs) globally [1]. Depressive disorders account for over 40% of DALYs for MNS disorders, with another 10% due to alcohol use disorders and 7% from psychosis. These estimates have increased by 15%, from 2005 to 2015, mostly due to ageing populations [1]. The paucity of available treatment for affected individuals is a major contributor to this burden. Different studies have estimated a mental health treatment gap of between 50 and 85%, with higher estimates found in low-income countries and for severe mental disorders [2, 3]. Emerging evidence provides support for the effectiveness and cost-effectiveness of treatment provision for several MNS disorders in low- and middle-income country (LMICs) settings [4–7]. This has formed the basis for the WHO mental health Gap Action Programme (mhGAP) guidelines on detection and treatment of MNS disorders by primary care providers [8]. Despite the availability of evidence-based treatment guidelines, actual implementation is a major challenge and there is a need to evaluate whether integrated care can reduce the burden of disability for adults affected by MNS disorders.

The aim of the Programme for Improving Mental Health Care (PRIME) consortium was to implement and evaluate district-level mental health care plans (MHCPs) in five LMIC settings [9] for four priority mental disorders: depression, alcohol use disorder (AUD), psychosis and epilepsy. The MHCP were informed by rigorous formative research and a participatory engagement with stakeholders [10]. Typically, these programmes include the identification of a MNS disorders and the provision of evidence-based mental health care by general health care providers at the primary care level, an approach known as task-sharing [11].

The evaluation of the MHCPs was carried out at the level of the district, community, facility and patient, using a range of methodologies based on a theory of change framework [12]. The impact of the MHCPs on clinical, functional and economic outcomes at the patient level was assessed through cohorts of adults identified with priority mental disorders, treated through the MHCPs and followed-up over time. The aim of the PRIME cohorts is to evaluate the implementation of the MHCPs on patient-level outcomes, and demonstrate whether task-shared, evidence-based treatments can be implemented at scale in LMIC settings to reduce the burden of disability for adults affected by MNS disorders.

A broad overview of the PRIME evaluation designs, including the cohort studies, has been published previously [12]. The aim of this paper is to provide a more detailed description of the cross-country methods and the methodological variations in each country site.

## Methods

### Objectives

The primary objective of the PRIME cohort studies is to assess the impact of the MHCPs on disability and symptom severity of adults diagnosed with a priority MNS disorder. Secondary objectives include 1) assessing change in productivity, economic status, stigma and discrimination (including for caregivers); 2) assessing health equity (e.g. by comparing the processes and outcomes of care by sex and socioeconomic status); and 3) identifying predictors of treatment effects.

### Study design

The cohort protocol was developed within an evaluation framework based on the Medical Research Council (MRC) framework for complex interventions [13], and using a Theory of Change approach [12]. This allowed cross-country research questions and resulting methods to be developed and implemented, while allowing for variation in local priorities. Based on the priority disorders included in the districts’ MHCPs, separate cohorts were recruited, one for each priority mental disorder. Participants for the depression and psychosis cohorts were recruited across all districts, while the epilepsy and AUD cohorts only comprised participants from selected districts.

### Study setting: mental health care plans

The PRIME cohort study took place in the following five low- and middle-income districts: Sodo (Ethiopia), Sehore (India), Chitwan (Nepal), Dr. Kenneth Kaunda (Dr KK) (South Africa), and Kamuli (Uganda). The MHCPs were developed according to the local needs and contexts of each district, and influenced by its geographical, social and cultural profile [14]. These have been described in detail [15–19]. Briefly, In Ethiopia, India, Nepal and Uganda, the four districts MHCPs adapted the mhGAP Intervention Guidelines [20] for local contextual needs and available resources. In South Africa, the MHCP uses an integrated set of chronic care guidelines called Adult Primary Care (APC, previously PC101), that has been adopted by the South African government and that incorporates mental health to initiate collaborative care [21].

All MHCPs comprised intervention packages at the community, health facility and health service organisation levels [15–19]. The community level packages typically included components relating to raising awareness, reducing stigma and discrimination, detecting and referring probable cases, as well as ongoing care, adherence support and rehabilitation. The facility-level packages included training and supervision to improve providers’ awareness, detection and psychosocial and/or psychotropic interventions for patients with a diagnosis of a priority disorder, and referrals to community or more specialised care. Finally, health service organisation level packages included aspects such as ensuring reliable supply of psychotropic medication, mechanisms for monitoring, capacity building and resource mobilisation.

All MHCPs also included basic psycho-education for all patients with a priority disorder diagnosis, across all districts. A basic or advanced psychosocial intervention was also offered to patients with depression and/or AUD, sometimes concurrently with medication, depending on symptom severity. In Nepal, a randomised controlled trial was embedded in the cohort study: half of the patients with depression or AUD received a basic psychosocial intervention from health workers, which included emotional support and psycho-education; the other half received an advanced evidence-based psychosocial intervention from non-professional community counsellors – a behavioural activation based intervention (depression) [22] or motivational activation intervention (AUD) [23].

Psychotropic medication was prescribed for patients with epilepsy and psychosis, with ongoing care and adherence support provided at community level in Ethiopia, South Africa and Uganda. More advanced psychosocial treatment, in the form of family counselling was also provided to patients with psychosis and epilepsy at community level by community counsellors in Nepal. In South Africa, the MHCP also included the provision of a rehabilitation group intervention to patients with a diagnosis of psychosis whose condition was stable.

### Participant eligibility

Table 1 provides an overview of the inclusion criteria and recruitment methods used across the districts and cohorts. Eligible participants had to meet the district’s MHCP criteria for treatment initiation, had to be above the country’s age of majority, be residents in the district, fluent in the local language and willing to provide informed consent. Patients with acute psychotic symptoms or who were not capable of providing consent were not eligible in any of the districts. In Ethiopia, however, consent from the guardian or caregiver was acceptable if the patient did not express objection to taking part in the study. Having been diagnosed with an MNS disorder prior to PRIME did not preclude patients from being eligible for enrolment in the psychosis and epilepsy treatment cohorts. While patients already receiving treatment for depression or AUD at the time of recruitment were not eligible for enrolment in Nepal and South Africa, they were eligible for enrolment in Ethiopia, India or Uganda.

**Table 1:** Recruitment and data collection method for the PRIME cohorts

	Sodo district, Ethiopia	Sehore district, India	Chitwan district^a^, Nepal	Dr Kenneth Kaunda, SA	Kamuli district, Uganda
District population	143,507 (total) [70]	318,314 (total) [71]	579,984 [72]	695,933 [73]	490,255 (total) [74]
Number of clinics involved in recruitment	9 facilities (8 health centres, 1 hospital)	3 community health centres	10 clinics	4 clinics	13 facilities (12 health centres, 1 hospital)
Recruitment period					
Depression	Feb 2015 – Dec 2015	Nov 2014 – July 2015	Aug 2014 – Sept 2015	Aug 2014 – July 2015	Jan 2015 - Sept 2015
AUD	Aug 2015 – Nov 2015	Nov 2014 – Aug 2015	Aug 2014 – Sept 2015	–	–
Psychosis	Dec 2014 – Jul 2015	Nov 2014 – Aug 2015	Aug 2014 – Sept 2015	Aug 2014 – Sept 2014 Aug 2015 – Sept 2015	Jan 2015 - Sept 2015
Epilepsy	Dec 2014 – March 2015	–	Aug 2014 – Sept 2015	–	Jan 2015 - Sept 2015
Step 1 of recruitment – Detection of individuals with priority mental disorder					
Depression	1. Diagnosis by mhGAP-trained nurse or health officer at clinic (MHCP)	1. mhGAP master chart checklist (MHCP) at community or clinic 2. PHQ-9 & AUDIT by case manager (MHCP), or else researcher, at clinic 3. Consultation with medical officer (MO) at clinic (MHCP)	1. Community informant detection tool (CIDT), at community (MHCP) 2. PHQ-9 & AUDIT by researcher, at clinic 3. Consultation with PHC worker or medical officer (MO), at clinic (MHCP)	1. Consultation with PC101 trained nurse or doctor, at clinic (MHCP) 2. PHQ-9 & AUDIT by researcher, at clinic	1. Consultation with mhGAP trained nurse or medical clinical officer, at clinic (MHCP)
AUD	1. Single-question alcohol screening test by mhGAP-trained nurse or health officer at clinic (MHCP) 2. AUDIT by mhGAP-trained nurse or health officer at clinic (MHCP)			–	
Psychosis	1. Identification of probable cases by HEWS and community key informant at community level (MHCP) 2. mhGAP master chart checklist by mhGAP-trained nurse or health officer used to identify psychosis or bipolar disorder (MHCP) 3. Confirmatory clinician interview (OPCRIT) by psychiatric nurse (MHCP)	1. mhGAP master chart checklist, at community or clinic (MHCP) 2. Consultation with MO, at clinic (MHCP)	1. Community information detection tool (CIDT), at community (MHCP) 2. Consultation with PHC worker or MO, at clinic (MHCP)	1.Identified from patient registry	
Epilepsy	1. Identification of probable cases by HEWS and community key informant at community level (MHCP) 2. mhGAP master chart checklist by mhGAP-trained nurse or health officer (MHCP) used to identify epilepsy 3. Diagnostic accuracy checked by neurologist in sub-sample of 25.	–		–	
Step 2 of recruitment – recruitment and group allocation					
Depression	Recruitment done by PRIME researcher; Group allocation: • Diagnosis made by nurse or health officer: diagnosed cohort • No diagnosis but screen positive on PHQ-9: comparison cohort	Recruitment done by PRIME researcher; Group allocation: • Diagnosis made by MO: diagnosed cohort • No diagnosis but screen positive on PHQ-9 or AUDIT: depression or AUD comparison cohorts	Recruitment done by PRIME researcher; Group allocation: • Diagnosis made by PHC worker: diagnosed cohort • No diagnosis but screen positive on PHQ-9 or AUDIT: depression or AUD comparison cohorts	Recruitment done by PRIME researcher; Group allocation: • Diagnosis made by nurse or doctor: diagnosed cohort • No diagnosis but screen positive on PHQ-9: comparison cohort	Recruitment done by PRIME researcher; • Group allocation: Diagnosis made by nurse: diagnosed cohort • No participants recruited in the comparison cohort
AUD	Diagnosis and recruitment done by PRIME researcher; • Screen positive on AUDIT: diagnosed cohort • No participants recruited in a comparison cohort			n/a	n/a
Psychosis	Diagnosis and recruitment done by psychiatric nurse; Diagnosed patient recruited, together with caregiver	Recruitment done by PRIME researcher; Diagnosis made by MO: diagnosed patient or caregiver recruited	Recruitment done by PRIME researcher; Diagnosis made by trained PHC worker or MO: caregivers of diagnosed patients recruited	Recruitment done by PRIME researcher: patient recruited; where possible, caregiver also recruited	Recruitment done by PRIME researcher; Diagnosis made by nurse: diagnosed patient recruited, together with caregiver
Epilepsy	Diagnosis and recruitment done by nurse or health officer; Diagnosed patient recruited, together with caregiver	n/a	Diagnosis given by PHC worker or MO: diagnosed patient recruited	n/a	
Assessments					
Location and timing of baseline assessment	All cohorts: Facility-based; if participants too unwell to leave their home, completed at home	All cohorts: Initiated at facility, finalised at home	All cohorts: Initiated at facility, finalised at home	All cohorts: Facility-based	Depression: Facility or home-based (depending on participant availability). Psychosis and epilepsy: Facility-based for participant, home-based for caregiver, or vice versa.
Location and timing of midline assessment	• Facility-based - if participants too unwell to leave their home, completed at home • Depression, psychosis and epilepsy: 6 months post-baseline • AUD: 3 months post-baseline	• Home-based • Depression and AUD: 3 months post-baseline • Psychosis: 6 month post-baseline	• Home-based • Depression and AUD: 3 months post-baseline • Psychosis and epilepsy: 6 month post-baseline	• Facility/Home-based • Depression: 3 months post-baseline • Psychosis: no midline	• Home-based • Depression: 3 months post-baseline • Psychosis and epilepsy: 6 month post-baseline
Location and timing of endline assessment	• Facility-based - if participants too unwell to leave their home, completed at home; • 12 months post-baseline	Home-based; 12 months post-baseline		• Facility/Home based; 12 months post-partum	Home-based; 12 months post-baseline

^a^The implementation area includes 10 of the 36 Village Development Committees in Chitwan District

PHC=Primary health care; PHQ-9 = Patient Health Questionnaire – 9 item; AUDIT = Alcohol Use Disorder Identification Test; OCPRIT = Operational Criteria Checklist for Psychotic Illness and Affective Illness; BRPSE = The Brief Psychiatric Rating Scale expanded version

The caregivers of patients with epilepsy or psychosis were also recruited either with or in lieu of the patient (see Table 1). A caregiver, identified by the patient with a disorder, was defined as the adult who was primarily responsible for meeting the daily needs of the patient. In Ethiopia, South Africa and Uganda, when possible, both the patient with psychosis and their caregiver were recruited, whereas in India, either one or the other were recruited depending on the patient’s ability to participate and complete the interview, determined by the trained data collectors. If the patient showed signs of being disoriented, having distorted communication or being unable to respond to questions, the patient was deemed unable to participate, and the caregiver was recruited and interviewed instead. In Nepal, caregivers were recruited and interviewed on behalf of patients diagnosed with psychosis, regardless of the patients’ ability to complete the interview. In Ethiopia and Uganda districts, caregivers were also recruited, in addition to the patients with epilepsy.

In certain districts, patients were recruited into depression or AUD comparison cohorts if they screened positive for depressive or AUD on a screening instrument, but were not diagnosed with depression or AUD by a PHC staff member, and therefore not eligible for treatment initiation as per the MHCP. Inclusion criteria were otherwise the same as for patients recruited into the depression and AUD treatment cohorts. More details on this procedure is described below.

### Recruitment process

Participants were recruited from primary health care clinics implementing the MHCPs in each district. Recruitment was conducted in several stages, again depending on how the MHCP operated. A description of how individuals were detected in each district and each cohort is provided below, as well as how, when and by whom they were recruited.

#### Community-based case detection

Three of the districts had community-based case detection included in their MHCP, with identification and referral of individuals with probable priority mental disorder in the community, either by community members or community health workers. In Ethiopia, this took the form of recognition of possible cases by health extension workers and community key-informants, trained in vignettes comprising typical presentations of psychosis and epilepsy. These vignettes had been used in a previous study for case ascertainment in a neighbouring district [24]. In Nepal, community members (Female Community Health Volunteers and mother groups) used a Community Informant Detection Tool (CIDT; [25]), specifically developed for the purpose of proactive identification of individuals in the community and to enhance help-seeking behaviours. The tool also makes use of vignettes and pictures to help lay individuals recognise relevant symptoms. In India, the mhGAP master chart checklist was used to detect probable cases in the community, which is based on the mhGAP guidelines. Detection was undertaken by mental health case managers, who were appointed as an additional human resource to facilitate the identification of individuals with priority mental disorders.

#### Facility-based recruitment for common mental disorders (depression and AUD)

All participants recruited in the depression and AUD treatment cohorts were diagnosed by a primary health worker, and all were screened with the Patient Health Questionnaire (PHQ-9; [26]) and/or the Alcohol Use Disorder Identification Test (AUDIT; [27]), before or after their consultation. In general, participants who received either a diagnosis of depression or AUD were recruited in the depression or AUD treatment cohorts. Participants who did not receive a diagnosis but screened positive on the PHQ-9 or AUDIT, were recruited into comparison cohorts (Table 1).

Specifically, in Ethiopia and Nepal, patients attending the primary care facilities were screened by PRIME researchers, using the PHQ-9, to identify potential participants to enrol into the depression treatment cohort. The screening was done before the patients’ consultation with a trained PHC worker or medical officer (MO)^1^ in Nepal, and after the consultation with a trained nurse or health officer in Ethiopia. PRIME researchers then followed-up patients (Nepal) or the PHC staff (Ethiopia) after the consultation to determine whether a diagnosis of depression was made. In Ethiopia, participants were only recruited into the depression comparison cohorts in the last two months of recruitment. Before that, only patients who screened positive and were diagnosed by the PHC staff were recruited into the depression treatment cohort.

The same process of recruitment applied for the AUD treatment cohort treatment and comparison cohorts in Nepal, but this time using the AUDIT as a screening tool. In Ethiopia, patients were only recruited into the AUD treatment cohort using a stepped approach: the nurse or health officer first used a single-question alcohol screening test [28] to identify patients at risk of AUD. If at risk, the AUDIT was then administered by the same health provider, and patients screening positive were recruited into the AUD treatment cohort by the PHC workers.

In South Africa, participants were recruited from the chronic care units in four primary health care clinics. Recruitment into the depression treatment cohort followed the same logic and process as in Ethiopia and Nepal. However, while patients were approached by the PRIME researchers for consent and recruitment before their consultation with the nurse or doctor, they were only screened with the PHQ-9 after their consultation. If patients were diagnosed with depression by a doctor, or identified with depression by a nurse,^2^ they were allocated to the depression treatment cohort, regardless of the screening scores. If patients had not been diagnosed/identified with depression but screened positive on the PHQ-9, they were recruited into the depression comparison cohort. If a diagnosis of depression was made at a subsequent facility visit, participants in the comparison cohorts were re-enrolled in the treatment cohort, and previous data deleted.

In India, recruitment was conducted after the consultation with the MO, but screening could be performed in one of two ways: 1) by the case managers at the clinic, after patients were suspected of having depression or AUD based on the mhGAP master chart checklist (at community or at the clinic), and prior to their consultation with the MO; or 2) by PRIME researchers, after the consultation with the MO, when screening was not conducted by the case managers (due to lack of time or because screened negative on the mhGAP master chart checklist). Similarly to Nepal and South Africa, if participants were diagnosed with AUD or depression by the MO in India, participants were allocated to the cohort treatment group. If they were not given a diagnosis but screened positive on the PHQ-9 or the AUDIT (regardless of who conducted the screening), the participants were allocated to the depression or AUDIT comparison cohorts, respectively. However, while the PHC worker or MO in Nepal were masked to the results of the screening scores, these were made available to the MO in India, to assist with diagnosis. In India, as in Nepal, priority was given to AUD in case of dual diagnosis or when participants screened positive on the PHQ-9 and AUDIT.

Finally, in Uganda, eligible patients were approached and enrolled by PRIME researchers on the day they received a diagnosis by a trained nurse or medical clinical officer, based on the mhGAP guidelines. No participants were recruited into the comparison cohorts, and the PHQ-9 was administered as part of the baseline assessment, after enrolment into the depression treatment cohort.

#### Facility based recruitment for psychosis and epilepsy

All patients recruited in the psychosis and epilepsy treatment cohorts, besides those recruited in South Africa had to have been diagnosed with the disorder by a PHC staff – an MO (India and Nepal), a psychiatric nurse (Ethiopia) or by a nurse (Uganda). In some instances, identification and recruitment was performed in a stepped manner.

In Ethiopia, the mhGAP master chart checklist was used to identify patients at risk of psychosis or bipolar disorder, and a clinician interview was then conducted by a psychiatric nurse to confirm the diagnosis, using the Operational Criteria Checklist for Psychotic Illness and Affective Illness (OPCRIT) [29]. The mhGAP master chart was also used to identify patients with epilepsy, however confirmation of diagnosis was conducted by a neurologist for a sub-sample of 25 patients. Once diagnosis was confirmed, the recruitment of participants in the psychosis and epilepsy treatment cohort was done by a mhGAP-trained nurse or health officer.

In India and Nepal, a suspected diagnosis of psychosis, either based on the mhGAP master chart checklist (India) or based on diagnosis by the PHC worker (in both districts), excluded patients from other cohorts, regardless of their screening scores on the PHQ-9 and AUDIT. The reason for this is that, in cases of comorbidity, priority for treatment (and cohort allocation) was given to the severe mental disorder, over CMDs.

In Uganda, participants were recruited by PRIME researchers into the psychosis and epilepsy treatment cohorts only once they were diagnosed by a trained nurse, based on the mhGAP guidelines.

Finally, in South Africa, participants with psychosis were identified from the clinic mental health patient register and approached to participate in the study. They had already been diagnosed with psychosis at a district/tertiary hospital, were considered stable, and had been referred back to primary health care for ongoing symptom management. Diagnosis was not re-confirmed before recruitment.

### Outcome measures

#### Primary outcomes

The cross-country sections or instruments included in the baseline and follow-up assessments for each cohort are presented in Table 2. The primary cross-country outcome for all cohorts was functioning, measured using the 12-item WHO Disability Assessment Schedule (WHODAS 2.0). Disorder-specific cross-country primary outcomes comprised clinical severity measures: number of seizures for epilepsy, the AUDIT score for AUD, and the PHQ-9 score for depression. The availability of specialist trained assessors limited the use of a psychosis-specific severity measures to the Brief Psychiatric Rating Scale – Expanded version (BPRS-E; [30]) in Ethiopia and South Africa, and the Positive and Negative Syndrome Scale (PANSS) in Nepal. The PHQ-9 was also collected as a secondary outcome for the AUD, psychosis and epilepsy treatment cohorts, given the comorbidity between depression and AUD [31], and between depression and severe mental disorders, including psychosis and epilepsy [32, 33]. The instruments used to measure the primary outcomes are described below.

**Table 2:** Assessment schedule for the PRIME cohorts

Data collected by questionnaire	Depression			Alcohol use disorders			Psychosis			Epilepsy		
Months of follow-up^a^	0	3/6	12	0	3	12	0	6	12	0	6	12
Demographics characteristics	✓			✓			✓			✓		
Clinical Measures												
WHO Disability Assessment Schedule (WHODAS 2.0) [34]	✓	✓	✓	✓	✓	✓	✓	✓	✓	✓	✓	✓
Patient Health Questionnaire (PHQ-9) [26]	✓	✓	✓	✓	✓	✓	✓	✓	✓	✓	✓	✓
Alcohol Use Disorder Identification Test (AUDIT) [27]	✓			✓	✓	✓	✓	✓	✓	✓		
Short Inventory of Problems – Recent (SIP 2-R] [75, 76]				✓	✓	✓						
Suicidality (Composite International Diagnostic Interview - suicidality module) [77]	✓	✓	✓	✓	✓	✓	✓	✓	✓	✓	✓	✓
Epilepsy severity (developed by PRIME)										✓	✓	✓
Brief Psychiatric Rating Scale (BPRS-E) [30]							✓	✓	✓			
Positive and Negative Syndrome Scale (PANSS) [78]							✓	✓	✓			
Health Service Use												
Group/community interventions (developed by PRIME)	✓	✓	✓	✓	✓	✓	✓	✓	✓	✓	✓	✓
Mental health services received (developed by PRIME)		✓	✓		✓	✓		✓	✓		✓	✓
Health Service use and costs (adapted from the Client Service Receipt Inventory)	✓	✓	✓	✓	✓	✓	✓	✓	✓	✓	✓	✓
Medication adherence												
Morisky Medication Adherence Scale (4-item) [79]	✓	✓	✓	✓	✓	✓	✓	✓	✓	✓	✓	✓
Medication adherence (adapted from Care for People with Schizophrenia in India)	✓	✓	✓	✓	✓	✓	✓	✓	✓	✓	✓	✓
Social and economic measures												
Economic activity (adapted from WHODAS 2.0, added items by PRIME)	✓	✓	✓	✓	✓	✓	✓	✓	✓	✓	✓	✓
Severe Adverse Events (developed by PRIME)		✓	✓		✓	✓		✓	✓		✓	✓
Oslo 3-item Social Support Scale [80]	✓	✓	✓	✓	✓	✓	✓	✓	✓	✓	✓	✓
Caregiver work burden - WHO Family Interview Schedule (Impact) [81]							✓	✓	✓	✓	✓	✓
Caregiver economic activity (adapted from WHODAS 2.0, items added by PRME)							✓	✓	✓			
Stigma and discrimination												
Discrimination and Stigma Scale [82]							✓		✓	✓		✓
Caregiver stigma & discrimination - WHO Family Interview Schedule (Stigma) [81]										✓		✓
Human rights abuse by caregiver (developed by PRIME)							✓	✓	✓	✓	✓	✓

^a^6 months for depression in Ethiopia

#### WHO disability assessment schedule

Disability was assessed using the WHODAS 2.0 (12 or 36 items) [34], an instrument developed by WHO and which has been validated in a range of settings and cultures [35], including India [36], South Africa [35] and Ethiopia [37]. The WHODAS was also previously used in studies conducted in Ethiopia [38], Nepal [39, 40] and Uganda [41]. The ‘item-response-theory’ (IRT) based scoring was used, and is suggested to facilitate comparisons across populations [34].

#### Patient Health Questionnaire (PHQ-9)

The PHQ-9 [26] is a widely used screening tool for depression among LMICs [42], and has previously been validated in primary health care patients in South Africa [43] and in India [44]. It was also recently validated in the Ethiopia [45], Uganda [46], Nepal [47] and South Africa [48] as part of the PRIME study. A cut-off of 10 was used by all districts to identify probable cases of depression, apart from Ethiopia, where a cut-off of 5 was found to be more culturally appropriate [45].

#### The Alcohol use Disorder Identification Test (AUDIT)

The AUDIT is a 10-item screening tool to identify alcohol misuse, developed by WHO [27]. The AUDIT has been validated in a range of settings [49]. It was shown to have good psychometric properties among HIV-positive individuals in outpatient care in South Africa [50], and was a valid and reliable measure in identifying dependent and hazardous drinkers in Eastern Nepal [51] and in New Delhi and Bangalore in India [52, 53]. Amharic and Luganda versions of the AUDIT have not yet been validated, but the AUDIT was found to have acceptable internal consistency among HIV-positive individuals in South West Ethiopia [54] and Southwestern Uganda [55]. Per WHO guidelines, the units of alcohol consumption for each item were locally contextualised. Different cut-offs were used to identify individuals with probable AUD: 8 in Ethiopia and India, and 9 in Nepal.

#### Brief psychiatric rating scale – extended version (BPRS-E)

The BPRS-E is a 24-item tool used to assess change in psychiatric symptoms among individuals with severe mental disorders, such as bipolar disorder and schizophrenia [30]. It is used in both clinical and research settings [56, 57]. Though used to assess severity of symptoms in the psychosis treatment cohort in Ethiopia and in South Africa, the reliability of the BPRS-E has not been assessed in these two countries. However, it has previously been used in both countries [58, 59], and evidence has generally shown the instrument to have a similar structure across different countries and settings [60].

#### Positive and Negative Syndrome Scale (PANSS)

Due to lack of clinical capacity to conduct the clinical-rated BPRS-E, the 14-item PANSS [61] symptom checklist was used in Nepal to assess positive and negative symptom severity among participants recruited in the psychosis treatment cohort. The 14 items have 5 response options, ranging from ‘never’ to ‘continuously’. The PANSS’s reliability has not previously been assessed in Nepal, but has been used successfully in previous research conducted in India [61].

### Secondary outcomes

A range of secondary outcomes for the participants were collected at all assessment points, comprising economic and health care expenditure measures, as well as stigma and discrimination measures. Secondary outcomes for the caregivers recruited in the psychosis and epilepsy treatment cohorts were also assessed, and these included caregiver work burden and stigma (Table 2).

Most secondary measures were standardised and already validated in similar settings. Some sections of the assessments, however, were developed or modified by the PRIME team to answer specific questions, such as clinical history, human rights abuse, or mental health treatment or community-based interventions received. Several secondary outcomes were optional, and each country also included country-specific sections; for this reason, assessments varied slightly across districts.

### Data collection

Recruitment and data collection were initiated between 6 and 12 months after the start of the MHCP implementation in each district, which included training staff, setting up supervision and leadership processes, as well as sensitisation activities. This allowed for the services to run for at least several weeks before recruitment started.

The baseline assessment was conducted following enrolment into the different cohorts. In India and Nepal districts, baseline assessments were initiated at the clinic and finalised in the participants’ home. The time elapsed between the enrolment of patients and the completion of the baseline interview could not exceed 7 days, and was completed on average 1 day after enrolment in Nepal, and after 3 days in India. In all other districts, the baseline assessment was conducted all at once, at the clinic or at the participants’ homes, on the day of diagnosis or enrolment (for comparison cohorts).

Despite the different methods of recruitment into the different cohorts across the districts, an attempt was made to retain cross-country consistency in the data collection methods. All participants in the cohorts were followed-up twice after the baseline assessment: for the AUD and depression treatment cohorts, the first follow-up was conducted three months after recruitment (+/− 2 weeks), except in Ethiopia where the first follow-up occurred at 6 months; for the psychosis and epilepsy treatment cohorts, the midline assessment was conducted 6 months after recruitment (+/− 2 weeks). A later follow-up assessment time for these two priority conditions was planned in anticipation of needing more time for patients to respond to treatment. The final follow-up was conducted 12 months after recruitment for all cohorts (+/− 4 weeks). Follow-up assessments were generally conducted in a private space at the participants’ home, though follow-up assessments were conducted at the clinic in Ethiopia and South Africa if more convenient for the participant.

An android mobile device application (Mobenzi; https://www.mobenzi.com) was used by interviewers to administer questionnaires for all districts, besides Ethiopia, where data were collected with paper and pencil and double entered in Epidata [62]. The use of Mobenzi, which allowed item skips and real-time scoring, meant that assessments were completed more quickly, with reduced human error and limited missing or unnecessary data. The baseline assessment took on average 1 h to administer on a Mobenzi device. Follow-up assessments, which excluded certain sections such as demographics or clinical history, were shorter and lasted approximately 30 min. In Ethiopia, where data was collected manually, assessment generally took longer to administer: approximately 1 h for the depression and AUD assessments, and approximately 2.5 and 2 h for the psychosis and epilepsy assessments, respectively.

Participants who could not be reached within the window period after at least three contact attempts were considered suspended until the next assessment, when an attempt to contact them was made again. Participants who actively refused to be assessed at follow-up were withdrawn from the cohort study. The reason for suspending participants from follow-up was recorded. This did not, however, affect the care they were receiving as part of the MHCP. Participants who, on the other hand, refused or discontinued treatment remained in the cohort and were still followed-up for their assessments.

### Statistical analyses

#### Power calculations

Sample sizes were calculated for each cohort, based on a 20% reduction in severity of symptoms at 12 months, with 90% power, two-sided alpha of 0.05, and 0.5 intra-class correlation. The initial sample size calculation was based on a one-sample analysis, so a one sample t-test power calculation was performed – this provided sample sizes between 30 and 70 depending on the standard deviation and instrument used for assessing symptom change, based on previous studies reporting pre-post screening scores [63–68]. However, an attrition rate of 15–20% at the end of the study was expected. Also, a bigger sample was required to be able to evaluate equity of the treatment effects (e.g. by gender and by socioeconomic status), to identify predictors of treatment effect, and to detect rare adverse events. The target sample size for cohorts in each district was therefore set at 200 for depression and AUD, and 150 for psychosis and epilepsy. The achieved sample sizes for each cohort are reported in Table 3.

**Table 3:** PRIME cohort sample sizes and attrition over time, by disorder and by district

	Depression		AUD		Psychosis		Epilepsy	
	Treatment	Comparison	Treatment	Comparison	Patient	Caregiver^a^	Patient	Caregiver
Ethiopia								
Enrolled	92	39	51	–	300	300	304	304
Attrition at midline	10 (10.9%)	0 (0%)	1 (2.0%)		53 (17.7%)	53 (17.7%)	149 (49.0%)	149 (49.0%)
Attrition at endline	13 (14.1%)	2 (5.1%)	4 (7.8%)	–	55 (18.3%)	55 (18.3%)	50 (16.4%)	50 (16.4%)
India								
Enrolled	281	158	218	147	22	21^b^	–	–
Attrition at midline	39 (13.9%)	15 (9.6%)	27 (12.3%)	19 (12.9%)	4 (19.0%)	0 (0%)		
Attrition at endline	56 (19.9%)	19 (12.1%)	43 (19.6%)	29 (19.7%)	4 (19.0%)	1 (5.0%)	–	–
Nepal								
Enrolled	137	72	175	57	–	95	42	–
Attrition at midline	27 (19.7%)	23 (31.9%)	40 (22.9%)	29 (50.9%)		8 (8.4%)	2 (4.8%)	
Attrition at endline	26 (19.0%)	17 (23.6%)	33 (18.8%)	22 (39.3%)	–	9 (9.5%)	4 (9.5%)	–
South Africa								
Enrolled	217	236	–	–	47	12	–	–
Attrition at midline	24 (11.1%)	27 (11.4%)			34 (72.3%)	8 (66.7%)		
Attrition at endline	40 (18.4%)	41 (17.3%)		–	5 (10.6%)	2 (11.1%)	–	–
Uganda								
Enrolled	64	–	–	–	51	50	181	171
Attrition at midline	3 (4.7%)				4 (7.8%)	6 (12.0%)	8 (4.4%)	8 (4.7%)
Attrition at endline	7 (10.9%)	–	–	–	8 (15.7%)	12 (24.0%)	19 (10.5%)	24 (14.0%)

^a^Caregivers recruited together with patient, unless otherwise stated

^b^Either patient or caregiver recruited

#### Primary and secondary analyses

The primary analyses of the PRIME cohort studies is to estimate the changes in disability and symptom severity over time among patients diagnosed with a priority mental disorder who initiated mental health care as part of the district MHCP. Secondary analyses include estimating change in productivity, economic status, stigma and discrimination; equity of primary and secondary outcomes; and identifying predictors of change in primary and secondary outcomes. Given the diversity of methods of recruitment used across districts, analyses will be stratified by district.

For change in continuous outcomes (e.g. WHODAS, PHQ-9, AUDIT), one-sample t-test and linear regression will be used when outcomes are normally distributed, to assess change from baseline to midline, and from baseline to endline. For skewed continuous outcomes, Poisson or negative binomial regression will be used instead, or in the case of extremely skewed outcomes, a non-parametric alternative, such as the Wilcoxon sign ranked test. For change in binary outcomes (e.g. suicidality), analyses will be conducted using logistic regression. The primary and secondary outcome analyses will be stratified by sex and then by socioeconomic status (lowest wealth to highest wealth) to assess equity of outcomes. Where there is evidence of heterogeneity, the stratum-specific effect estimates will be presented. Additional equity analyses will be considered by each district (e.g. by caste in Nepal, and by rural vs. urban residence in Ethiopia).

As mentioned above, four districts also recruited a depression and AUD comparison cohort. Two sample t-tests and linear, Poisson or negative binomial regressions will be used to estimate the difference-in-differences of outcome means at each time point in comparison to the baseline. Baseline imbalances in sociodemographic and clinical measures between the treatment and comparison cohorts in each cohort will be adjusted in the models, if feasible. Also, when possible, multiple imputation methods will be used to adjust estimates for loss to follow-up.

Potential factors associated with primary and secondary outcomes at each follow-up visit will be assessed using linear (or Poisson or negative binomial) regression and logistic regression, for continuous and binary outcomes, respectively.

### Ethical considerations

This study was approved by the University of Cape Town’s Health Sciences Faculty Human Research Ethics committee (HREC REF: 412/2011), South Africa, and by the WHO Research Ethics Review Committee, Switzerland. Each district also received Ethical approval from their relevant local Research Ethics Committees. Consent forms were translated in local languages and completed by all participants who agreed to participate, and/or by their caregivers, where appropriate. However, patients did not need to provide permission for caregivers to be recruited. Refusing to take part in the cohort study or discontinuing participation after enrolment did not prevent patients from receiving clinical care as part of the MHCP.

## Discussion

### Practical and operational issues

The practical and operational issues that arose during recruitment and data collection in the PRIME treatment cohorts, and how these issues were dealt with, are outlined below.

### Sample size and attrition

The final sample size and follow-up rates of each cohort in each district are reported in Table 3. The recruitment of participants into the cohorts took longer than expected, and was a function of the low detection rate by primary health care staff, as described in the MHCP [15–19]. Refresher training sessions and continuous supervision to ensure PHC workers were still proactive with detection were put in place. Despite these efforts, and due to time constraints relating to other PRIME-related research activities, recruitment had to be discontinued before some of the cohorts could reach the optimal sample size.

Attrition rates were, for the most part, within the attrition range expected, and accounted for by the increased sample size (Table 2.). Among participants recruited in the treatment cohorts, attrition generally ranged between 4 and 20% for the midline assessment, and between 10 and 20% at the endline assessment, across districts and cohorts. Migration was the main reason for loss to follow-up for all cohorts. Particularly high attrition rates for depression and AUD comparison cohorts were reported in Nepal, where 76.4 and 60.7% completed the endline assessment, respectively. The primary reasons for non-completion in these groups were participants no longer wanting to take part in the study (35 and 23%, respectively), or moving away from the district (47 and 50%, respectively). Given that these participants were not receiving care under the MHCPs, it is understandable that they were perhaps more difficult to retain in the study, compared to participants in the treatment groups.

Fewer midline assessments were conducted for the psychosis treatment cohort in South Africa, since the start of the 12-session group rehabilitation intervention was delayed. This delay meant that not all participants had completed the intervention by the end of the midline assessment window. When this was the case, the midline assessment was skipped and only a 12-month (endline) follow-up was conducted. Limited resources also meant that only half of the participants in the epilepsy treatment cohort could be followed-up for their midline assessment in Ethiopia.

### Comparison cohorts

It was not feasible or ethical, given the study was taking place in routine settings, to create a ‘regular’ control group where the MHCPs were not implemented. For this reason, though not ideal, a comparison cohort of non-diagnosed individuals who screened positive on the PHQ-9 or the AUDIT were also recruited in Ethiopia, India, Nepal and South Africa. These comparison participants provide an approximation of the trajectory of outcomes for the treatment cohort participants, had the latter not received treatment. There may, however, be systematic differences between treatment and comparison cohort participants which may limit our ability to make conclusive estimates of the treatment effects, even after these are controlled for statistically.

Many of the difficulties encountered in the recruitment and data collection procedures for the PRIME cohorts emerged from the tension between research processes and the implementation of mental health services as part of the MHCP in the districts. This is especially reflected in the relatively small samples sizes recruited for some cohorts, timing of assessments in relation to the completion of the treatment prescribed, and the inability, ethically, to recruit diagnosed but untreated patients into comparison cohorts. However, a rigorous process was involved in identifying measures to include in the assessment over time, based on the Medical Research Council complex intervention framework [13] and the Theory of Change [69]. Meaningful indicators of change were identified, as well as the relevant locally-validated tools and instruments to assess these indicators. This meant we could assess a wide range of outcomes (i.e. functional, clinical, social and economic), thereby providing a holistic perspective of patient recovery. So, while the heterogeneity in methods of recruitment largely reflected the differences in health systems and MHCPs, the use of common standardised tools should also allow for comparability across sites. Finally, the process and outcome measures collected as part of the cohort study will enable us to identify which elements of the districts’ MHCPs were implemented properly, which should be revised, and which are necessary for success outcomes for individuals with MNS disorders.

## Abbreviations

AUD: Alcohol use disorder
AUDIT: Alcohol use disorder identification test
BPRS-E: Brief psychiatric rating scale extended version
CIDT: Community informant detection tool
CMD: Common mental disorders
Dr. KK: Dr. Kenneth Kaunda
LMICs: Low- and middle-income countries
MHCP: Mental health care plan
mhGAP: Mental health treatment gap
MNS: mental, neurological and substance use
MO: Medical officer
OCRPIT: Operational criteria checklist for psychotic illness and affective illness
PANSS: Positive and negative syndrome scale
PHQ-9: Patient health questionnaire – 9 item
PRIME: Programme for improving mental health care
WHO: World Health Organization
WHODAS: WHO disability assessment schedule

## Footnotes

1 Medical officers are considered doctors in Nepal and India – they are recent graduates of medical school.

2 Nurses were trained to detect depression but could not make a formal diagnosis. However, they had the authority to refer patients for psychosocial counselling and for recruitment into the depression treatment cohort. When medication was necessary, patients had to be diagnosed and prescribed medication by a medical doctor.
