# Correction to: Impact of district mental health care plans on symptom severity and functioning of patients with priority mental health conditions: the Programme for Improving Mental Health Care (PRIME) cohort protocol

**DOI:** 10.1186/s12888-020-02890-9

**Published:** 2020-09-29

**Authors:** Emily C. Baron, Sujit D. Rathod, Charlotte Hanlon, Martin Prince, Abebaw Fedaku, Fred Kigozi, Mark Jordans, Nagendra P. Luitel, Girmay Medhin, Vaibhav Murhar, Juliet Nakku, Vikram Patel, Inge Petersen, One Selohilwe, Rahul Shidhaye, Joshua Ssebunnya, Mark Tomlinson, Crick Lund, Mary De Silva

**Affiliations:** 1 Alan J Flisher Centre for Public Mental Health, Department of Psychiatry and Mental Health, University of Cape Town, 46 Sawkins Road 7700 Rondebosch, Cape Town, South Africa; 2 Department of Population Health, London School of Hygiene and Tropical Medicine, London, UK; 3 College of Health Sciences, School of Medicine, Department of Psychiatry, Addis Ababa University, Addis Ababa, Ethiopia; 4 Centre for Global Mental Health, Health Services and Population Research Department, King’s College London, London, UK; 5 Centre for Innovative Drug Development and Therapeutic Trials for Africa, College of Health Sciences, Addis Ababa University, Addis Ababa, Ethiopia; 6 Global Health and Infection Department, Brighton and Sussex Medical School, University of Sussex, Brighton, UK; 7 Butabika National Referral and Teaching Mental Hospital, Makerere University, Kampala, Uganda; 8 Research and Development Department, HealthNet TPO, Amsterdam, the Netherlands; 9 Center for Global Mental Health, Institute of Psychiatry, Psychology and Neuroscience, King’s College London, London, UK; 10 Research Department, Transcultural Psychosocial Organization (TPO) Nepal, Baluwatar, Kathmandu, Nepal; 11 Aklilu Lemma Institute of Pathobiology, Addis Ababa University, Addis Ababa, Ethiopia; 12 Sangath, Goa, India; 13 Harvard Medical School, Boston, USA; 14 Public Health Foundation of India, New Delhi, India; 15 Centre for Rural Health, School of Nursing and Public Health and School of Applied Human Sciences, University of KwaZulu-Natal, KwaZulu-Natal, South Africa; 16 Centre for Chronic Conditions and Injuries, Public Health Foundation of India, New Delhi, India; 17 CAPHRI School for Public Health and Primary Care, Maastricht University, Maastricht, the Netherlands; 18 Alan J Flisher Centre for Public Mental Health, Department of Psychology, Stellenbosch University, Stellenbosch, South Africa; 19 Department of Population Health, Wellcome Trust, London, UK

**Correction to: BMC Psychiatry 18, 61 (2018)**

**https://doi.org/10.1186/s12888-018-1642-x**

Following publication of the original article [1], the authors identified an error in Table 2. The correct table is given below.

**Table 2:** Assessment schedule for PRIME Cohorts

Data collected by questionnaire	Depression			Alcohol use disorders			Psychosis			Epilepsy		
Months of follow-up*	0	3/6	12	0	3	12	0	6	12	0	6	12
DEMOGRAPHICS CHARACTERISTICS	✓			✓			✓			✓		
CLINICAL MEASURES												
WHO Disability Assessment Schedule (WHODAS 2.0) [34]	✓	✓	✓	✓	✓	✓	✓	✓	✓	✓	✓	✓
Patient Health Questionnaire (PHQ-9) [26]	✓	✓	✓	✓	✓	✓	✓	✓	✓	✓	✓	✓
Alcohol Use Disorder Identification Test (AUDIT) [27]	✓			✓	✓	✓	✓	✓	✓	✓		
Short Inventory of Problems – Recent (SIP 2-R] [75, 76]				✓	✓	✓						
Suicidality (Composite International Diagnostic Interview - suicidality module) [77]	✓	✓	✓	✓	✓	✓	✓	✓	✓	✓	✓	✓
Epilepsy severity (developed by PRIME)										✓	✓	✓
Brief Psychiatric Rating Scale (BPRS-E) [30]							✓	✓	✓			
Positive and Negative Syndrome Scale (PANSS) [78]							✓	✓	✓			
HEALTH SERVICE USE												
Group/community interventions (developed by PRIME)	✓	✓	✓	✓	✓	✓	✓	✓	✓	✓	✓	✓
Mental health services received (developed by PRIME)		✓	✓		✓	✓		✓	✓		✓	✓
Health Service use and costs (adapted from the Client Service Receipt Inventory)	✓	✓	✓	✓	✓	✓	✓	✓	✓	✓	✓	✓
MEDICATION ADHERENCE												
Morisky, Green and Levine Adherence Scale [79]	✓	✓	✓	✓	✓	✓	✓	✓	✓	✓	✓	✓
Medication adherence (adapted from Care for People with Schizophrenia in India)	✓	✓	✓	✓	✓	✓	✓	✓	✓	✓	✓	✓
SOCIAL AND ECONOMIC MEASURES												
Economic activity (adapted from WHODAS 2.0, added items by PRIME)	✓	✓	✓	✓	✓	✓	✓	✓	✓	✓	✓	✓
Severe Adverse Events (developed by PRIME)		✓	✓		✓	✓		✓	✓		✓	✓
Oslo 3-item Social Support Scale [80]	✓	✓	✓	✓	✓	✓	✓	✓	✓	✓	✓	✓
Caregiver work burden - WHO Family Interview Schedule (Impact) [81]							✓	✓	✓	✓	✓	✓
Caregiver economic activity (adapted from WHODAS 2.0, items added by PRME)							✓	✓	✓			
STIGMA AND DISCRIMINATION												
Discrimination and Stigma Scale [82]							✓		✓	✓		✓
Caregiver stigma & discrimination - WHO Family Interview Schedule (Stigma) [81]										✓		✓
Human rights abuse by caregiver (developed by PRIME)							✓	✓	✓	✓	✓	✓

* 6 months for depression in Ethiopia
